# Agonist-Antagonist Coactivation Enhances Corticomotor Excitability of Ankle Muscles

**DOI:** 10.1155/2019/5190671

**Published:** 2019-09-03

**Authors:** Trisha M. Kesar, Andrew Tan, Steven Eicholtz, Kayilan Baker, Jiang Xu, Joanna T. Anderson, Steven L. Wolf, Michael R. Borich

**Affiliations:** ^1^Division of Physical Therapy, Department of Rehabilitation Medicine, Emory University, Atlanta, Georgia, USA; ^2^Department of Physical Medicine and Rehabilitation, Harvard Medical School and Spaulding Rehabilitation Hospital, USA; ^3^Rollins School of Public Health, Emory University, Atlanta, Georgia, USA; ^4^Department of Rehabilitation Medicine, Tongji Hospital, Tongji Medical College, Huazhong University of Science and Technology, Wuhan, China; ^5^Department of Physiology, Emory University, Atlanta, Georgia, USA; ^6^Center for Visual and Neurocognitive Rehabilitation, Atlanta Veterans Affair Health Care System, Decatur, GA, USA

## Abstract

Spinal pathways underlying reciprocal flexion-extension contractions have been well characterized, but the extent to which cortically evoked motor-evoked potentials (MEPs) are influenced by antagonist muscle activation remains unclear. A majority of studies using transcranial magnetic stimulation- (TMS-) evoked MEPs to evaluate the excitability of the corticospinal pathway focus on upper extremity muscles. Due to functional and neural control differences between lower and upper limb muscles, there is a need to evaluate methodological factors influencing TMS-evoked MEPs specifically in lower limb musculature. If and to what extent the activation of the nontargeted muscles, such as antagonists, affects TMS-evoked MEPs is poorly understood, and such gaps in our knowledge may limit the rigor and reproducibility of TMS studies. Here, we evaluated the effect of the activation state of the antagonist muscle on TMS-evoked MEPs obtained from the target (agonist) ankle muscle for both tibialis anterior (TA) and soleus muscles. Fourteen able-bodied participants (11 females, age: 26.1 ± 4.1 years) completed one experimental session; data from 12 individuals were included in the analysis. TMS was delivered during 4 conditions: rest, TA activated, soleus activated, and TA and soleus coactivation. Three pairwise comparisons were made for MEP amplitude and coefficient of variability (CV): rest versus coactivation, rest versus antagonist activation, and agonist activation versus coactivation. We demonstrated that agonist-antagonist coactivation enhanced MEP amplitude and reduced MEP CVs for both TA and soleus muscles. Our results provide methodological considerations for future TMS studies and pave the way for future exploration of coactivation-dependent modulation of corticomotor excitability in pathological cohorts such as stroke or spinal cord injury.

## 1. Introduction

Transcranial magnetic stimulation (TMS) is a noninvasive brain stimulation technique that can be used to evaluate descending corticomotor drive to ankle muscles during both static [1] and dynamic tasks [2]. The amplitude of motor-evoked potentials (MEPs) elicited in response to TMS reflects the cumulative excitability of the corticospinal pathway [3, 4]. Due to differences in the anatomy and physiology of neural control circuits controlling limb muscles, there is a need to evaluate methodological factors influencing TMS-evoked MEPs specifically in lower limb musculature [5, 6]. A key confound to using TMS-evoked MEP amplitudes as a measure of primary motor cortex (M1) excitability is their sensitivity to concurrent changes in the excitability of subcortical and segmental spinal circuitry [7, 8]. Additionally, TMS-evoked MEPs may modulate due to the activation of targeted muscles of the leg contralateral to [9–12] as well as activation of homologous muscles in the leg ipsilateral to the stimulated hemisphere [13]. Recent work from our lab and others has shown that the postural state also influences TMS-evoked MEP amplitude of leg muscles [14–16]. However, if and to what extent the activation of the nontargeted muscles, such as antagonists, influences TMS-evoked MEP output is poorly understood, and such gaps in our knowledge may limit the rigor and reproducibility of TMS studies focused on the lower limb.

Neural control of agonist and antagonist muscles (e.g., ankle dorsiflexors and plantar flexors) is organized reciprocally, such that the activation of the agonist is accompanied by simultaneous inhibition of the antagonist muscle. During activation of the tibialis anterior (TA), H-reflexes from the antagonist muscle (soleus) are depressed [17]. Compared to rest, during TA muscle activation, there is an increase in disynaptic spinal reciprocal inhibition from the TA to soleus motoneurons, mediated by Ia interneurons [17]. Interestingly, in contrast to this reciprocal inhibition of antagonist motoneurons within spinal segmental circuitry, TMS-evoked MEPs from the soleus have been shown to be facilitated during [12] and before [7] voluntary dorsiflexion. Thus, while there is increase in reciprocal inhibition of the soleus motoneuron and depression of soleus H-reflexes during TA activation, cortically evoked soleus MEPs may show facilitation during TA activation [7]. Geertsen et al. suggest that during functional tasks requiring rapid modulation of movement direction, voluntary contraction at the ankle is preceded by facilitation of antagonists, likely mediated by subcortical motor programs. While spinal disynaptic pathways underlying reciprocal ankle flexion-extension contractions have been extensively characterized, the extent to which changes in cortical excitability or descending drive, as captured by MEP amplitude, are influenced by antagonist muscle activation remains unclear.

Experiments in both nonhuman primates [18, 19] and humans [20, 21] provide evidence for specialized descending control of agonist-antagonist coactivation versus isolated flexion and extension movements. During coactivation, spinal reciprocal inhibition between antagonistic ankle muscles is depressed, likely through descending suppression of Ia interneurons. During agonist-antagonist coactivation, an upregulation in coactivation-specific descending drive modulates spinal segmental circuitry and may influence the TMS-induced MEP amplitude. Coactivation of agonist and antagonist muscles is an important component of normal motor control. Coactivation is a key feedforward strategy used to regulate joint stiffness [22] and increase joint impedance in response to external perturbations [23] for tasks that demand high accuracy [24] or when joint stability is compromised [25, 26]. Tasks that require coactivation-specific motor programs, such as standing on an unstable platform, are accompanied by decreases in reciprocal inhibition [27] and simultaneous reductions in reflex amplitude [25, 28]. Additionally, increased agonist-antagonist coactivation during functional tasks is common motor control abnormality observed in individuals with neuropathologies such as stroke [29, 30]. Thus, using TMS to study changes in corticomotor excitability during an ankle coactivation task provides an opportunity to gain insights into neuromotor control of lower limb muscles.

Relatively sparse evidence explicitly characterizes how antagonist muscle coactivity influences TMS-evoked MEPs. For example, Geertsen et al. demonstrated that voluntary contraction at the ankle is accompanied by preceding facilitation of antagonists, likely mediated by subcortical circuitry [7]. Modulation of the excitability of 1a inhibitory interneurons, alpha motoneurons, and subcortical sites of descending inhibition can markedly influence how the TMS-elicited descending volley activates the final common pathway (lower motoneuron pool), as well as the size of MEP evoked from the target muscle during TMS. TMS studies commonly record and monitor background activation and MEPs only from the target muscle. Yet, synergist and antagonist muscle activation can modulate the excitability of supraspinal and spinal segmental circuitry and influence TMS-evoked MEP responses of the target muscle. Understanding how the activation state of the antagonist muscle affects TMS-evoked MEP amplitude can inform the interpretation and design of TMS studies. The purpose of this study, therefore, was to evaluate the effect of the activation state of the antagonist muscle on TMS-evoked MEPs obtained from the target (agonist) ankle muscle for both TA and soleus muscles.

## 2. Methods

The study procedures were approved by the Emory University Institutional Review Board. All participants provided informed written consent in accordance with the Declaration of Helsinki.

### 2.1. Participants

Nineteen young, neurologically intact subjects with no history of orthopedic or neurological conditions were enrolled in this study. Exclusion criteria included previous or current neurologic disorders and contraindications to TMS including history of seizure, metal implants in the head, recent concussion, use of central nervous system-acting drugs, pregnancy, or episode of syncope or loss of consciousness in the past 12 months [31]. Five of the 19 participants did not complete the study protocol or were excluded due to contraindications to TMS, including a syncope incident [32], medications, previous concussion, and dizziness/discomfort during TMS. The remaining 14 participants (11 females, age: 26.1 ± 4.1 years) completed the study protocol.

### 2.2. Study Design

Data reported were collected during a single session, as part of a larger study investigating the effect of muscle activation, posture, and walking tasks on lower limb cortical excitability.

#### 2.2.1. Electromyography Procedures

After standard skin preparation procedures to minimize skin impedance, bipolar, circular, and self-adhesive surface EMG sensors (11 mm diameter, 11 mm interelectrode distance, BIOPAC Systems Inc., USA) were attached to the skin overlying the belly of the right TA and soleus muscles, with a common ground sensor attached over the right lateral malleolus [33]. EMG sensor placement was confirmed by checking the EMG signal during voluntary muscle contraction. EMG data were sampled at 1000 Hz, band-pass filtered from 10 Hz to 500 Hz, and amplified by 2000 (AcqKnowledge software, BIOPAC Systems Inc.). To maintain consistent sensor location throughout the session and minimize EMG movement artifact, EMG sensors and wires were wrapped securely to the leg (Sensi-Wrap, Dynarex Inc.).

#### 2.2.2. EMG Activity during Maximum Voluntary Contractions (MVCs)

EMG data were collected while the participant performed 3 maximal voluntary contractions (MVCs) of 5 s duration of the right soleus and TA muscles. For the soleus MVC, the participants were instructed to stand on their right foot and plantar flex their ankle by lifting the heel off the ground. For the TA MVC, the participant maximally dorsiflexed their ankle against resistance while in a seated position. A 3-second window during peak contraction was used to calculate the average root mean square EMG amplitude, and the maximal value of the 3 contractions was identified as the MVC EMG. The MVC EMG was used to set the low-level target background activation level (10% MVC) that the participants would maintain during TMS assessments.

#### 2.2.3. TMS Procedures

Single-TMS pulses were delivered using a custom, “batwing” figure-of-eight coil, which has a slightly different angulation and shape than the double-cone coil (70 mm diameter, Magstim Company Ltd., UK) connected to a monophasic stimulator (Magstim 200^2^). Stereotaxic neuronavigation (Brainsight, Rogue Research Inc., Canada) was used to track the 3-dimensional position and orientation of the coil, with the participant's head coregistered to a standard template brain (MNI 152) throughout the experiment. During TMS, the coil was held tangential to the scalp with the coil handle parallel to the interhemispheric fissure, in order to induce a posteroanterior current within the cortex. The hotspot for the right TA muscle within the contralateral (left) primary motor cortex (M1) was determined as the optimal coil position that elicited maximal MEP responses from the right TA at the lowest stimulator intensity [34]. For each participant, coil position and orientation at the hotspot was saved and used as a reference throughout the session. Using a computerized adaptive algorithm [35], the resting motor threshold (rMT) was determined with an MEP amplitude criterion of 80 *μ*V [34]. The criterion MEP amplitude for rMT was increased from 50 *μ*V [34] to 80 *μ*V to ensure that discernible MEPs would be obtained at rest from both TA and soleus muscles. For the remaining experimental protocol, TMS intensity was maintained at the same suprathreshold intensity (120 or 130% of rMT) for all testing conditions.

#### 2.2.4. EMG Biofeedback for Maintaining Consistent Background EMG Activation

Raw EMG signals from the right TA and soleus were sent as analog outputs from the BIOPAC hardware to a data acquisition board (USB-6343 X-Series, National Instruments) and input into a custom EMG biofeedback program (LabVIEW, National Instruments) to calculate the root mean square (RMS) EMG for the TA and soleus and to visually display the RMS EMG for the participant in real time. When the testing condition required that one muscle be activated (e.g., soleus activated), biofeedback was provided to the participant to help maintain the specific muscle's activation within the target EMG window (10% MVC, tolerance range = ±5%). When the testing condition required that both muscles be activated (e.g., coactivation), biofeedback was provided for both muscles simultaneously. EMG biofeedback ensured that participants maintained desired background EMG activation levels for each condition and that background EMG magnitude for a muscle (e.g., TA) was consistent across testing conditions (e.g., between TA activation and coactivation).

#### 2.2.5. Data Collection during Different TMS Testing Conditions

**Figure 1:**
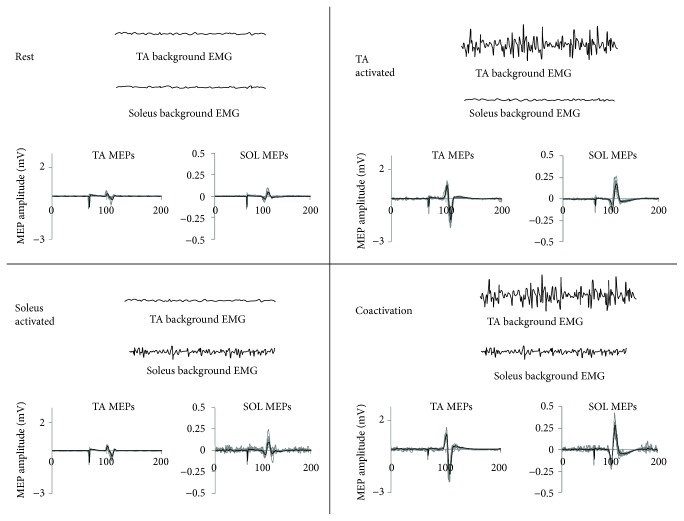
The four activation conditions evaluated in the current study. The schematic shows the 4 conditions and exemplary raw background EMG data traces for the TA and soleus muscles. Additionally, for each of the 4 conditions, raw TA and soleus MEP data are shown for a representative study participant (gray lines represent individual MEPs and black lines represent averaged MEP).

A custom-written software program (AcqKnowledge, BIOPAC Systems Inc.) was used to deliver 10-15 TMS pulses at 0.2 Hz. Data acquisition was delineated so that 50 ms of data were collected before and 450 ms were collected after the TMS pulse, with a total acquisition time of 500 ms for each TMS pulse (Figure 1). Participants were seated comfortably in a chair with the back supported, neck unsupported, knee and hip at 90° flexion, and the ankle at 0° dorsiflexion. The alignment of the participant's feet was then marked with tape on a floor mat to ensure consistent foot placement during the session. An ankle weight was positioned over the dorsum for stabilization. TMS-evoked MEPs were collected during 4 testing conditions, in random order (Figure 1):

1. *Rest*: both TA and soleus were at rest
2. *TA activated*: the TA was activated and the soleus was at rest
3. *Soleus activated*: the soleus was activated and the TA was at rest
4. *Coactivation*: both the TA and soleus muscles were activated

For all testing conditions, TMS was delivered with the coil over the hotspot of the right TA (determined at the start of the session) and MEPs were simultaneously recorded from the TA and soleus. For this study, the agonist muscle was the muscle targeted during TMS (i.e., the muscle from which TMS-evoked MEPs are being evaluated as a dependent variable). When evaluating TMS-evoked MEPs from the TA muscle, the agonist refers to TA and antagonist to soleus. When evaluating TMS-evoked MEPs for the soleus muscle, the agonist refers to soleus and antagonist to the TA.

During TA or soleus activation, participants were provided visual biofeedback about the ongoing EMG for the muscle that needed to be activated. Participants were instructed to either lift their foot up (dorsiflexion) or downward (plantar flexion) to increase ongoing EMG to the target value. During the coactivation condition, the participants were shown ongoing EMG for both TA and soleus on the EMG biofeedback screen and asked to try to activate each muscle to the target value. If needed, they were given verbal instructions such as “try to stiffen your ankle while pushing upward into the foot pad” to aid with achieving the coactivation task. Data from these 4 conditions were used to make 3 comparisons for each muscle (TA and soleus) and to test the following 3 hypotheses listed below. We did not compare rest to agonist activation because this has been well studied previously. The 3 comparisons are as follows:

Comparison (1) *Rest versus coactivation*: for both TA and soleus muscle MEPs, we hypothesized that compared to the resting state, TMS-evoked MEPs will be enhanced during coactivation
Comparison (2) *Rest versus antagonist activation*: for both TA and soleus muscle MEPs, we hypothesized that compared to rest, TMS-evoked MEPs will be facilitated during the antagonist activation condition
Comparison (3) *Agonist activation versus coactivation*: for both TA and soleus muscle MEPs, for similar background activation levels of the agonist (target) muscle, we hypothesized that TMS-evoked MEPs will be larger during agonist-antagonist coactivation compared to agonist activation alone

### 2.3. Data Analysis

Prestimulation EMG background RMS amplitude for both TA and soleus muscles was calculated for a 50 ms window prior to the delivery of TMS (Figure 1). Background EMG RMS data were reviewed by investigators for each comparison; trials where the subject's EMG did not match the condition being tested (evaluated using between-condition paired *t*-tests on individual subject's MEP trials) were removed to ensure that comparisons between different testing conditions were not confounded by differences in background EMG activity. The TA and soleus peak-to-peak MEP amplitude was calculated for each trial as the difference between the maxima and minima within the MEP window. For each subject, the mean peak-to-peak amplitudes for 10-15 MEP trials were used as a measure of corticospinal excitability during each testing condition. For each condition, the trial-to-trial variability in MEP amplitude was indexed by calculating the coefficient of variation (CV) (standard deviation of the MEP amplitudes divided by the mean MEP amplitude × 100).

### 2.4. Statistical Analysis

The primary dependent variables were MEP amplitudes for the TA and soleus muscles. Because variations in background EMG preactivation can influence the TMS-evoked MEPs, background EMG RMS data were checked for individual trials and participants, as well as evaluated as a secondary dependent variable. Additional secondary variables included trial-to-trial CV of MEP amplitude. We evaluated the assumption of normal distribution using the Kolmogorov-Smirnov test, which was met for our dependent measures with the exception of TA MEP amplitude for a couple conditions, and the background EMG RMS in the resting condition.

A repeated measures ANOVA was used to compare the primary variables (TA and soleus MEP amplitude) among the 4 testing conditions (rest, agonist on, antagonist on, and coactivation). Additionally, for each muscle, predetermined pairwise comparisons were conducted to make 3 specific comparisons: rest versus coactivation, rest versus antagonist activation, and agonist activation versus coactivation. Repeated 'measures 1-way ANOVA with post hoc paired *t*-tests was also conducted for secondary variables (background EMG RMS, CV of MEP amplitude).

Additionally, to systematically evaluate individual subject MEP amplitude data, for each paired comparison, change scores were calculated as the difference in MEP amplitude for the two comparison conditions (e.g., MEP amplitude during coactivation minus MEP amplitude during rest). MEP change scores were plotted for each participant and used to evaluate the interindividual variability of change scores and to assess whether the 95% confidence interval (CI) of the change score included zero [36]. Similarly, to check background EMG data, change scores for each comparison were computed and plotted for the background EMG RMS. Background EMG change scores were used to confirm that background EMG matched the comparison of interest and to exclude outliers, i.e., any participant with a change scores that exceeded 2.5 times standard deviation of the mean for that comparison.

To compare the magnitude of modulation of TMS-evoked MEPs induced by varying the testing conditions, for each muscle (TA and soleus), a repeated measures 1-way ANOVA with post hoc pairwise comparisons was performed to compare MEP change scores among the 3 study comparisons. The 3 change scores were coactivation‐rest (coactivation minus rest), antagonist‐rest (antagonist activation minus rest), and coactivation‐agonist (coactivation minus agonist activation). SPSS version 21 was used for statistical analysis. An alpha level was set at 0.05.

## 3. Results

Two of 14 participants who completed the study were excluded from all comparisons due to their background activation exceeding the instructed target value.

### 3.1. Overall Effects of the Antagonist Muscle Activation State

The repeated measures ANOVAs revealed a significant main effect of testing condition on both TA (*F* = 21.998; *p* < 0.001) and soleus MEP amplitudes (*F* = 15.045; *p* < 0.001). The ANOVA comparing trial-to-trial coefficient of variation (CV) of MEP amplitude across the 4 conditions showed a main effect of condition for TA (*F* = 10.859; *p* < 0.001) and soleus (*F* = 8.580; *p* < 0.001). The repeated measures ANOVAs revealed a significant main effect of testing condition on both TA (*F* = 57.621; *p* < 0.001) and soleus background EMG RMS amplitudes (*F* = 29.384; *p* < 0.001). The results for the post hoc pairwise comparisons are listed for each of the comparisons below.

### 3.2. Comparison between Rest and Agonist-Antagonist Coactivation

#### 3.2.1. TA MEP Amplitudes

**Figure 2:**
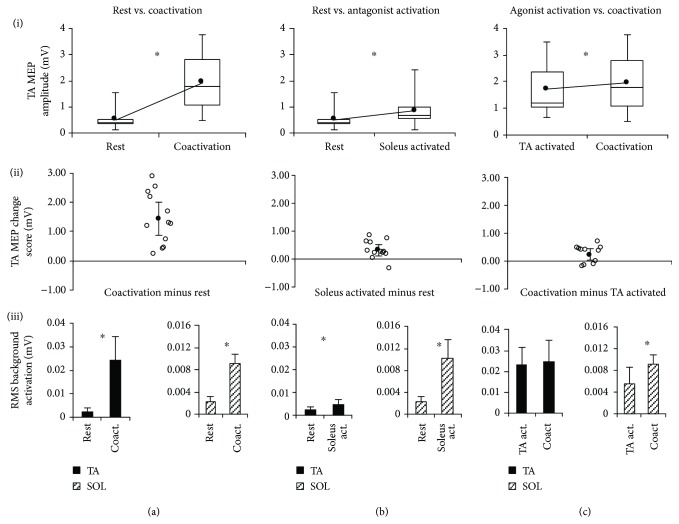
TMS-evoked MEP results for the TA muscle. The 3 columns of graphs show each of the 3 comparisons made in the current study for the TA muscle: rest versus coactivation (a), rest versus antagonist activation (b), and agonist activation versus coactivation (c). The 3 rows of graphs show 3 sets of data for each comparison: (i) MEP amplitudes, (ii) MEP change scores, and (iii) background EMG RMS value for both the agonist/target muscle (TA) and antagonist (soleus). In row (i), the box and whisker plots are shown for TA MEP amplitudes, with the group average of all subjects' TA MEPs shown by the line plot. In row (ii), the TA MEP change score scatter plots show each individual's change score as well as the average (group mean) change score with error bars representing the 95% CI of the mean. In row (iii), the RMS background activation for both TA and soleus muscles is plotted. ∗ indicates statistically significant differences (*p* < 0.05).

**Figure 3:**
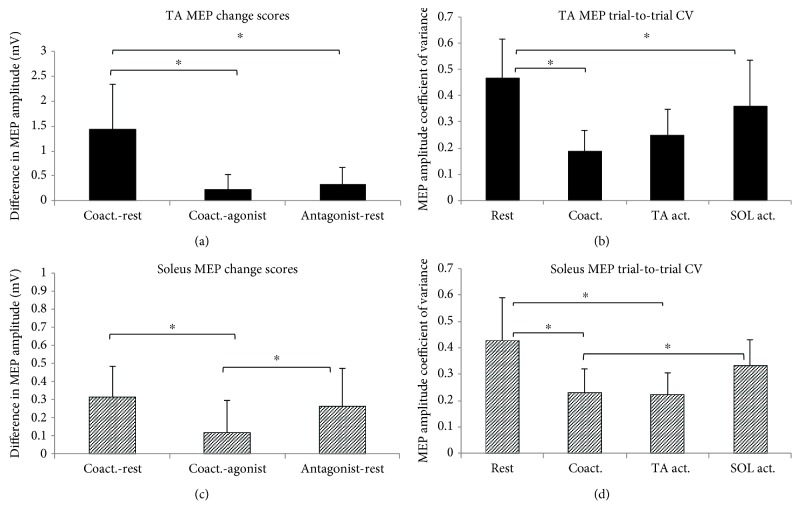
Effects of antagonist activation on change scores and trial-to-trial coefficient of variance of TA and soleus MEPs. The barplots on the left depict the between-condition MEP change scores for the TA (a) and soleus (c) muscles (means and standard errors across participants). For each muscle, 3 comparisons are depicted: Coact.‐rest (coactivation condition minus rest condition), Coact.‐agonist (coactivation condition minus agonist activation condition), and antagonist‐rest (antagonist activation condition minus rest condition). The plots on the right depict the trial-to-trial coefficient of variation (CV) of MEP amplitudes for the TA (b) and soleus (d) muscles (means and standard errors across all study participants). Of the comparisons conducted (e.g., comparison between rest and agonist activation was not of interest in the current study), post hoc comparisons that showed a statistically significant difference are indicated by the symbol ∗. Note that the agonist refers to the muscle of interest (i.e., for TA MEP data, TA is the agonist).

MEP amplitude for the TA was significantly larger (*p* < 0.001) during coactivation (1.950 ± 1.042 mV) compared to rest (0.516 ± 0.364 mV) (Figure 2(a), (i)). Individual participant change scores showed that all participants exhibited an increase in MEP amplitude during coactivation (positive change score) compared to rest and the 95% CI of the change score did not include zero (Figure 2(a), (ii)). There was a significantly greater (*p* < 0.001) trial-to-trial CV of MEP amplitudes during rest (46.5 ± 15.0%) compared to coactivation (18.8 ± 8.1%) (Figure 3(b)).

#### 3.2.2. Soleus MEP Amplitudes

**Figure 4:**
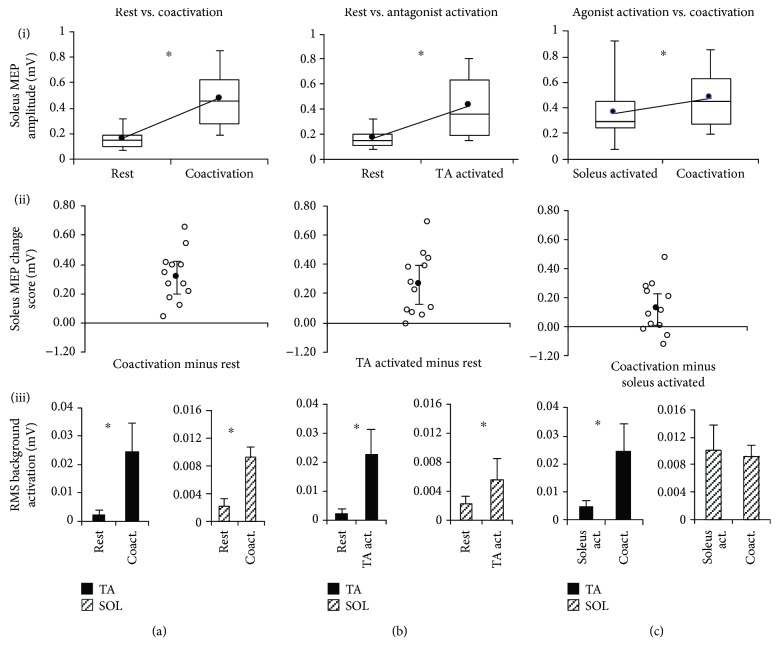
TMS-evoked MEP results for the soleus muscle. The 3 columns of graphs show each of the 3 comparisons made in the current study for the soleus muscle: rest versus coactivation (a), rest versus antagonist activation (b), and agonist activation versus coactivation (c). The 3 rows of graphs show 3 sets of data for each comparison: (i) MEP amplitudes, (ii) MEP change scores, and (iii) background EMG RMS value for both the agonist/target muscle (soleus) and antagonist (TA). In row (i), the box and whisker plots are shown for soleus MEP amplitudes, with the group average of all subjects' soleus MEPs shown by the line plot. In row (ii), the soleus MEP change score scatter plots show each individual's change score as well as the average (group mean) change score with error bars representing the 95% CI of the mean. In row (iii), the RMS background activation for both TA and soleus muscles is plotted. ∗ indicates statistically significant differences (*p* < 0.05).

Soleus MEP amplitude was significantly larger (*p* < 0.0001) during coactivation (0.477 ± 0.216 mV) compared to rest (0.166 ± 0.081 mV) (Figure 4(a), (i)). Individual subject change scores showed that all participants exhibited an increase in MEP amplitude during coactivation compared to rest and the 95% CI of the change score did not include zero (Figure 4(a), (ii)). There was a significantly greater (*p* = 0.005) trial-to-trial CV of soleus MEP amplitudes during rest (42.6 ± 16.2%) versus coactivation (23.0 ± 9.0%) (Figure 3(d)).

#### 3.2.3. Background Electromyography (EMG) Activation

The background EMG root mean square (RMS) amplitude for the TA was significantly lower (*p* < 0.001) during rest (0.0025 ± 0.0014 mV) compared to coactivation (0.0246 ± 0.0099 mV) (Figures 2(a), (iii) and 4(a), (iii)). Similarly, the background EMG RMS for the soleus was significantly lower (*p* < 0.001) during rest (0.0022 ± 0.0010 mV) compared to coactivation (0.0093 ± 0.0016 mV) (Figures 2(a), (iii) and 4(a), (iii)).

### 3.3. Comparison between Rest and Antagonist Activation

#### 3.3.1. TA MEP Amplitudes

TA MEP amplitudes were significantly larger (*p* = 0.005) during antagonist (soleus) activation (0.851 ± 0.609 mV) compared to rest (0.516 ± 0.364 mV) (Figure 2(b), (i)). Individual change scores for the TA MEPs showed that all subjects except one showed an increased MEP amplitude (positive change score) during antagonist activation versus rest, with the 95% CI not including zero (Figure 2(b), (ii)). There was a significantly greater (*p* = 0.030) trial-to-trial CV of MEP amplitudes during rest versus antagonist activation (Figure 3(b)).

#### 3.3.2. Soleus MEP Amplitudes

Soleus MEP amplitudes were significantly larger (*p* = 0.001) during antagonist (TA act.) activation (0.426 ± 0.242 mV) versus rest (0.166 ± 0.081 mV) (Figure 4(b), (i)). Individual change scores in the soleus MEP amplitude showed that a majority of the subject showed increased MEP amplitude during antagonist activation versus rest (positive change scores) with the 95% CI not including zero (Figure 4(b), (ii)). There was a significantly greater (*p* = 0.008) trial-to-trial CV during rest compared to antagonist activation (Figure 3(d)).

#### 3.3.3. Background EMG

There was a significant difference (*p* = 0.004) in the TA background EMG between rest (0.0025 ± 0.0014 mV) and antagonist (soleus) activation (0.0049 ± 0.0020 mV) (Figure 2(b), (iii)). There was a significant difference (*p* < 0.001) in the soleus background EMG between rest (0.0022 ± 0.0010 mV) and antagonist (soleus) activation (0.0101 ± 0.0035 mV) (Figure 2(b), (iii)).

There was a significant difference (*p* < 0.001) in the TA background EMG between rest (0.0025 ± 0.0014 mV) and antagonist (TA) activation (0.0229 ± 0.0085 mV) (Figure 4(b), (iii)). There was a significant difference (*p* = 0.003) in the soleus background EMG between rest (0.0022 ± 0.0010 mV) and antagonist (TA) activation (0.0056 ± 0.0029 mV) (Figure 4(b), (iii)).

### 3.4. Comparison between Agonist Activation and Coactivation

#### 3.4.1. TA MEP Amplitudes

TA MEP amplitudes were significantly larger (*p* = 0.020) during coactivation (1.950 ± 1.042 mV) versus agonist activation (1.714 ± 0.979 mV) (Figure 2(c), (i)). Individual change scores showed high intersubject variability; however, most subjects showed an increase in MEP amplitude during coactivation compared to agonist activation and the 95% CI did not include zero (Figure 2(c), (ii)). The TA MEP amplitude CV did not show a significant difference (*p* = 0.107) during agonist activation compared to coactivation (Figure 3(b)).

#### 3.4.2. Soleus MEP Amplitudes

Soleus MEP amplitudes were significantly larger (*p* = 0.044) during coactivation (0.477 ± 0.216 mV) versus agonist activation (0.361 ± 0.220 mV) (Figure 4(c), (i)). The individual change scores showed high intersubject variability, with the majority of subjects showing an increase in soleus MEP amplitude for coactivation compared to agonist activation, and the 95% CI did not include zero (Figure 4(c), (ii)). Soleus MEP CVs were significantly greater (*p* = 0.040) during agonist activation versus coactivation (Figure 3(d)).

#### 3.4.3. Background EMG

There was no difference (*p* = 0.156) in the TA background EMG between agonist (TA) activation (0.0229 ± 0.0085 mV) and coactivation (0.0246 ± 0.0099 mV) (Figure 2(c), (iii)). There was a significant difference (*p* < 0.001) in the soleus background EMG between agonist (TA) activation (0.0056 ± 0.0029 mV) and coactivation (0.0093 ± 0.0016 mV) (Figure 2(c), (iii)).

There was a significant difference (*p* < 0.001) in the TA background EMG between agonist (soleus) activation (0.0049 ± 0.0020 mV) and coactivation (0.0246 ± 0.0099 mV) (Figure 4(c), (iii)). There was no difference (*p* = 0.387) in the soleus background EMG between agonist (soleus) activation (0.0101 ± 0.0036 mV) and coactivation (0.0093 ± 0.0016 mV) (Figure 4(c), (iii)).

### 3.5. Comparison of Modulation in MEP Amplitude with Varying Antagonist Activation

The one-way repeated measures ANOVA evaluating the effect of the 3 comparison types on change in MEP amplitude showed a main effect of comparison type for both TA (*F* = 19.601; *p* < 0.001) and soleus (*F* = 8.171; *p* = 0.002) MEP change scores (Figure 3). Pairwise post hoc comparisons for the TA muscle showed that the largest change in MEP amplitude was observed for the transition from rest to coactivation. TA MEP change scores for coactivation-rest were significantly greater than change scores for antagonist-rest (*p* = 0.001) and coactivation-agonist (*p* < 0.001) (Figure 3(a)). Similarly, post hoc comparisons showed that for soleus MEPs, MEP change scores for coactivation-rest were significantly greater than antagonist-rest (*p* < 0.002) but not different from coactivation-agonist change scores (*p* = 0.12). There was a significant difference between antagonist-rest and coactivation-agonist for the soleus (*p* = 0.05) (Figure 3(c)).

## 4. Discussion

Our results showed that agonist-antagonist muscle coactivation significantly enhances TMS-evoked MEPs recorded from ankle muscles. For both TA and soleus, when compared to the condition when both muscles were at rest, agonist-antagonist coactivation resulted in larger MEP amplitudes. After matching the agonist muscle's background EMG activation between-conditions, the MEP amplitude was significantly greater during coactivation compared to agonist activation. Our findings suggest that during a volitional coactivation task, coactivation-specific descending drive and modulation of reciprocal inhibition enable the TMS-induced descending volleys to elicit larger MEPs. We further demonstrated that compared to rest, activating the antagonist muscle or engaging in agonist-antagonist coactivation resulted in smaller trial-to-trial variability (CV) of MEP amplitude. Thus, agonist-antagonist coactivation as well as antagonist activation may be feasible strategies to increase the probability of eliciting consistent and measurable MEPs from lower limb muscles, especially in neurologically impaired individuals who show elevated motor thresholds. Furthermore, our results underscore the importance of monitoring background activation from the antagonist muscle during TMS experiments.

Increases in the size of TMS-evoked MEP have been documented to parallel increases in volitional background activation of the target or agonist in both upper limb [37] and lower limb muscles [1]. However, there is sparse evidence regarding whether MEP amplitudes are influenced by antagonist coactivation. Here, we undertook a direct comparison of the effect of antagonist coactivity on agonist MEPs. A unique aspect of our approach was that we compared agonist-antagonist coactivation to rest as well as to agonist-only activation conditions. Our results showed that compared to rest, perhaps unsurprisingly, MEP amplitude of the target muscle increased during the coactivation condition. More importantly and interestingly, coactivation also induced a significant increase in MEP amplitude compared to the agonist activation condition, despite a nonsignificant between-condition difference in the agonist or target muscle's background activation level. Thus, taken together, both these comparisons provide strong support for our conclusion that agonist-antagonist coactivation enhances TMS-evoked MEP amplitude of the target or agonist muscle.

Activity-dependent modulation of spinal circuit excitability has been previously studied during tonic [21, 38] and dynamic tasks [17], as well as during walking [2]. Despite this characterization, few studies have used TMS to explicitly evaluate corticospinal excitability of an antagonist/agonist muscle pair during a volitional coactivation task. At the spinal segmental level, coactivation is regulated by different spinal mechanisms, including reciprocal inhibition [39] and presynaptic inhibition of Ia afferents [40]. However, at the cortical level, descending regulation of transmission within spinal Ia inhibitory interneurons plays a role in allowing coactivation of antagonist muscles [21, 41]. Potentially, certain corticospinal neurons may be coactivation-specific [18], yet the extent to which coactivation-dependent increases in TMS-evoked MEPs are reflective of an upregulation of excitability in coactivation-specific descending circuitry remains to be determined. While our methods and results cannot parse out the contribution of a specific neural control mechanism to ankle muscle coactivation, they provide unique evidence of increased corticospinal excitability that accompanies agonist-antagonist coactivation.

Previous suggestions about stronger descending input to the TA motoneuron pools [42] compared to soleus [43] lead to the question of whether antagonist activation or coactivation-dependent influences on MEP amplitude differ between the soleus and TA muscles. Geertsen et al. previously reported facilitation of soleus MEPs during antagonist contractions before and during movement initiation [7]. Interestingly, here, we observed a reciprocal effect in the increase in MEP during coactivation irrespective of whether the TA or soleus were functioning as the agonist or target muscle during TMS. During functional tasks involving the ankle, descending control specific to coactivation may depress transmission in reciprocal inhibition [44, 45] to allow coactivation of agonist and antagonist motoneuron pools. An uncoupling of descending input to Ia inhibitory interneurons and descending drive to motoneurons may facilitate coactivation tasks and appears to manifest for both TA and soleus muscles.

Other lines of evidence suggest differential cortical activation during coactivation versus agonist-only activation tasks. In addition, the activation of ankle muscles, both volitionally and in response to the TMS-induced volley, may not necessarily activate isolated motoneuron pools. Imaging studies reveal a distinct and larger pattern of M1 cerebral activation during coactivation compared to agonist-only or antagonist-only activation after matching EMG levels in isolated dorsi/plantar flexion [46]. However, while the increased activation may indicate increased utilization of a subset of coactivation-specific cortical networks, the cortical demand associated with task complexity may alternatively explain cortical activation intensity. Cortical activation has been linked to the demand [47] and type of motor task [46, 48]. The volitional coactivation task used here may be sufficiently complex to require greater cognitive effort and cortical activation and perhaps distinct descending circuits. Parsing out the influence of cognitive effort during coactivation or skilled motor tasks on TMS-evoked MEPs would be an interesting future study. The current data, unfortunately, cannot discern if the increased MEP amplitude during coactivation versus rest was caused by differences in attentional focus or motor task complexity.

Further characterization of the effect of coactivation between heteronymous pairs of muscles (e.g., soleus and quadriceps) may yield additional insight into the generalizability of the current results [49]. Systematic quantification of coactivation-dependent modulation of TMS-evoked MEPs across multiple lower limb muscles may help resolve specific cortical control of synergist and antagonist muscles. Additionally, recordings during dynamic tasks such as ramp and hold contractions and during other postural conditions such as standing may elucidate the task specificity of the current observations. Further study of coactivation-dependent MEP modulation may be especially pertinent during gait, where phase-specific modulation of corticospinal excitability [50] and reciprocal inhibition [2] supports gait-specific motor cortex involvement in the tuning of muscle coactivation.

From a methodological viewpoint, increasing corticomotor excitability of the agonist by modulating antagonist activation may be advantageous during evaluation of corticomotor excitability of lower limb muscles using TMS. For example, eliciting TMS-evoked MEPs from the paretic leg is particularly challenging in individuals with elevated motor thresholds after stroke. Employing targeted coactivation as a testing condition may remediate challenges associated with reduced corticomotor excitability in the paretic limb of persons with stroke, likely by gating specialized coactivation circuitry. Our present results, if observed in individuals poststroke, suggest that maintaining low-level coactivation of antagonist muscles can potentially lower activation thresholds and increase the probability of eliciting lower limb MEP responses. Furthermore, given the increasing importance of identifying the presence or absence of MEP as a prognostic tool in neurorehabilitation in people poststroke [51], alternate testing conditions such as the coactivation task that increase the probability of eliciting MEPs from ankle muscles can be advantageous. As another example, in a clinical scenario where a stroke survivor has greater weakness in TA than soleus and obtaining MEPs from the TA (target muscle) is not possible, our results suggest that the activation of the soleus (antagonist) may facilitate measurement of MEPs from the paralyzed TA muscle. This is especially salient for TMS investigations of persons with paresis in dorsiflexor or plantar flexor muscles, where the coactivation of the less impaired antagonist muscle may sufficiently augment the MEPs of the paretic muscle agonist [48, 52]. However, MEPs elicited using modified testing conditions (such as coactivation or antagonist activation) may offer different mechanistic insights or interpretations compared to MEPs elicited at rest. Along similar lines, during lower limb TMS, the activation of the contralateral limb muscles has also been shown to enhance TMS-evoked MEPs from the targeted ankle muscles [13]. Additionally, the lower trial-to-trial CV of MEP amplitudes demonstrated in our results during the coactivation condition provides another methodological advantage of using the coactivation condition in future investigations.

Our results also showed that compared to rest, the antagonist activation condition resulted in larger MEP amplitude for both soleus and TA muscles. However, this finding was limited by our observation that when instructed to activate the antagonist muscle only, study participants inadvertently increased agonist background activity as well. Thus, a small but significant magnitude of agonist activation was present during the antagonist activation condition. Due to this methodological limitation, we were unable to demonstrate a testing condition that represented “pure” antagonist-only activation condition. This low-level agonist activation that accompanied the task command to activate the antagonist may be due to methodological factors, e.g., we only provided EMG biofeedback for the muscle to be activated and we checked background EMG of the nonactivated muscle during post hoc data analysis and not during the experiment. Alternatively, unlike muscles of the hand or fingers, lower limb muscles may have greater propensity for coactivation as opposed to individuation. Notwithstanding the cause, this limitation affected our comparisons involving the antagonist activation condition and not the coactivation conditions.

Our study has several limitations. The target 10% of MVC required here likely does not approach an upper limit in the progressive motor unit recruitment where the probability of increased stimulus response to TMS sharply declines [53, 54]. While linear increases in MEP responses are observed to plateau around 50% MVC [37] in upper limb muscles, no decreases in MEP response were observed in the soleus across a wide range of contraction levels [1]. Unfortunately, in those studies, the levels of antagonist coactivation were not reported. The present results do not permit delineation of how higher levels of voluntary antagonist coactivation affect the stimulus response curve. Nonetheless, the current study provides preliminary evidence that corticospinal excitability to the agonist muscle is facilitated even by low force cocontractions of ankle muscles. Although we tested EMG sensor placement and checked for cross talk, cross talk can influence the study results. Similarly, varying the TMS stimulation intensity may affect the size of the evoked responses for both muscles. The TMS stimulus intensity was dosed to the TA resting motor threshold and not the soleus. A suprathreshold intensity was chosen to achieve consistent MEP responses from both the TA and soleus muscles for each condition. Although the TA and soleus hotspots likely overlap, the ability to selectively activate TA and soleus motor representations is confounded by their proximate locations within interhemispheric fissure and the limited focality of the TMS-induced magnetic field [6]. Given the spillover of TMS-induced electric field to nontargeted muscles, disassociating effects caused by generalized stimulation of lower limb cortex are difficult. Finally, the current study used TMS to evaluate changes in overall corticospinal tract excitability with the modulation of the antagonist muscle activation state; inclusion of measures of spinal excitability (e.g., TA-Soleus reciprocal inhibition) in future studies would provide additional insights about the site and mechanism of our findings. Moreover, because agonist muscle activation was not equivalent during the antagonist activation and coactivation conditions, these two conditions were not compared in our study.

## 5. Conclusions

The current study takes an important step towards clarifying the influence of the activation state of antagonist muscles during TMS and provides methodological considerations for future TMS evaluation of lower limb muscles. We demonstrated that antagonist activation as well as agonist-antagonist coactivation enhanced TMS-evoked MEP amplitude, while reducing trial-to-trial MEP variability, for both TA and soleus muscles. Our results suggest the need to explore the effect of coactivation-dependent modulation of corticomotor excitability in pathological cohorts such as stroke or spinal cord injury. Future TMS studies in combination with spinal reflex measurements may further elucidate the neural control of coactivation.

## Data Availability

The neurophysiologic data used to support the findings of this study are available from the corresponding author upon request.
